# Quantifying levels of biological invasion: towards the objective classification of invaded and invasible ecosystems

**DOI:** 10.1111/j.1365-2486.2011.02549.x

**Published:** 2011-10-27

**Authors:** Jane A Catford, Peter A Vesk, David M Richardson, Petr Pyšek

**Affiliations:** * School of Botany, The University of MelbourneVictoria, 3010, Australia; † Centre for Invasion Biology, Department of Botany & Zoology, Stellenbosch UniversityStellenbosch, South Africa; ‡ Institute of Botany, Academy of Sciences of the Czech RepublicPrůhonice, Czech Republic; § Department of Ecology, Faculty of Science, Charles University in PraguePrague, Czech Republic

**Keywords:** ecological indicators, ecosystem invasibility, functional groups, generalization, invasive alien species, nonnative animals and plants, standard metrics, transformer species

## Abstract

Biological invasions are a global phenomenon that threatens biodiversity, and few, if any, ecosystems are free from alien species. The outcome of human-mediated introductions is affected by the invasiveness of species and invasibility of ecosystems, but research has primarily focused on defining, characterizing and identifying invasive species; ecosystem invasibility has received much less attention. A prerequisite for characterizing invasibility is the ability to compare levels of invasion across ecosystems. In this paper, we aim to identify the best way to quantify the level of invasion by nonnative animals and plants by reviewing the advantages and disadvantages of different metrics. We explore how interpretation and choice of these measures can depend on the objective of a study or management intervention. Based on our review, we recommend two invasion indices and illustrate their use by applying them to two case studies. Relative alien species richness and relative alien species abundance indicate the contribution that alien species make to a community. They are easy to measure, can be applied to various taxa, are independent of scale and are comparable across regions and ecosystems, and historical data are often available. The relationship between relative alien richness and abundance can indicate the presence of dominant alien species and the trajectory of invasion over time, and can highlight ecosystems and sites that are heavily invaded or especially susceptible to invasion. Splitting species into functional groups and examining invasion patterns of transformer species may be particularly instructive for gauging effects of alien invasion on ecosystem structure and function. Establishing standard, transparent ways to define and quantify invasion level will facilitate meaningful comparisons among studies, ecosystem types and regions. It is essential for progress in ecology and will help guide ecosystem restoration and management.

## Introduction

Whether introduced species become established, naturalized or invasive is influenced by inherent features of the species and recipient ecosystems, and factors associated with human activities (Drake *et al*., 1989; Richardson & Pyšek, 2006). Considerable effort has been made to define, characterize and identify species at different stages of the introduction-naturalization-invasion continuum (Richardson *et al*., 2000; Colautti & MacIsaac, 2004; Pyšek & Richardson, 2007; Valéry *et al*., 2008; Blackburn *et al*., 2011). An **alien** (exotic, nonnative, nonindigenous, introduced) species is considered **invasive** when it sustains self-replacing populations over several life cycles, spreads considerable distance from its site of introduction and often reaches very large numbers (Richardson *et al*. 2000, 2011). Some general characteristics have emerged that distinguish invasive alien species from noninvasive ones (Daehler, 2003; Pyšek & Richardson, 2007; Nentwig *et al*., 2010; van Kleunen *et al*., 2010; Davidson *et al*., 2011), but much less attention has been devoted to quantifying and characterizing invasibility.

**Invasibility** is defined as the vulnerability of a habitat and the associated biological community to invasion (Alpert *et al*., 2000; Davis *et al*., 2000; Milbau *et al*., 2009). It is an emergent property of ecosystems (i.e. it has characteristics that differ from its constituent parts) and is affected by abiotic conditions such as climate, nutrient availability and disturbance, and features of the resident biota (Lonsdale, 1999; Richardson *et al*., 2000). Invasibility can be characterized by the survival rate of invading species when species identity and the number of individuals introduced are held constant. However, as propagule pressure differs among habitats and varies across the landscape (Lonsdale, 1999; Chytrý *et al*., 2008b; Eschtruth & Battles, 2011), and the identity and ability of species to invade also differs, the influence of species invasiveness and propagule pressure on invasion level must be accounted for when quantifying invasibility.

Some ecosystems are considered more invasible than others (Richardson *et al*., 2007; Chytrý *et al*., 2008b), but invasibility has rarely been quantified – perhaps because it requires information about invasion level, species invasiveness and propagule pressure, which is difficult to quantify (Eschtruth & Battles, 2011). Recent advances in identifying characteristics associated with invasiveness (Daehler, 2003; Nentwig *et al*., 2010; van Kleunen *et al*., 2010) and defining meaningful proxies for propagule pressure (Pino *et al*., 2005; Herborg *et al*., 2007; Chytrý *et al*., 2008a; Pyšek *et al*., 2010) hold the promise that invasibility can be quantified. A prerequisite for this is the ability to compare levels of invasion across ecosystems.

**Invasion level** refers to the extent or severity of alien invasion observed in an ecosystem (Chytrý *et al*., 2008a). A growing number of studies examine invasion levels among ecosystems and regions, but the metrics used are often not directly comparable (e.g. alien species richness: Lonsdale, 1999; Stohlgren *et al*., 1999; Deutschewitz *et al*., 2003; Stohlgren *et al*., 2003; Ortega & Pearson, 2005; relative alien species richness: Chytrý *et al*., 2008a, 2009; alien species cover: Ortega & Pearson, 2005; relative alien cover: Catford & Downes, 2010; Catford *et al*., 2011b). Like other ecological indices (Cairns *et al*., 1993), we see the purpose of an invasion level index as: (1) facilitating the assessment of the extent or severity of alien species invasion in an area; (2) revealing trends in invasion level through space and time, and – by revealing these trends; (3) acting as an early warning sign for ecological degradation.

Establishing a standard metric of invasion is not only essential for determining invasibility, but has inherent value. Invasion level scores could be used to gauge the following:

- the ecological consequences of invasion, e.g. biotic homogenization, competition with native species, alteration of ecosystem structure and disruption of ecosystem function, changes to biotic interactions and ecological networks like pollination and dispersal;
- economic costs of invasion, e.g. loss of ecosystem services, reduced agricultural production, cost of control interventions; and
- the potential for control, eradication and recovery postinvasion (Nicholson *et al*., 2009).

An index of invasion level can also be used to guide management efforts. If two ecosystems are invaded by a different suite of species, how can management resources be prioritized objectively if the net impact of invasion on the ecosystems is unknown? If Ecosystem A has twice as many native species as alien species, but alien species make up half the total plant cover abundance, is it more severely invaded than Ecosystem B that has the same numbers of native and alien species, but where alien cover is only 25% of total cover? Identifying standard, comparable metrics will help with such decisions (Colautti & Richardson, 2009).

In this paper, we aim to identify the best ways to quantify the level of invasion by alien animals and plants, and explore how the selection and interpretation of these measures can depend on the objective of a study or management intervention. We start by discussing the criteria by which ecological indicators should be selected. We then consider potential indicators of invasion level, including ways to quantify it and what sort of response variables are of interest. Based on our review, we recommend two invasion indices and illustrate their use by applying them to two case studies. We briefly discuss how invasion level can be used to gauge invasibility by accounting for propagule pressure and invader traits at the end of the paper. Establishing standard, transparent ways to define and quantify invasion level will facilitate meaningful comparisons among studies, ecosystem types and regions, and it provides a necessary step towards determining the invasibility of ecosystems.

## Selecting suitable indicators of invasion level

The choice of invasion indices and interpretation of their scores may differ depending on study objectives (Parker *et al*., 1999), area or units of interest (e.g. communities, habitats, regions) and current understanding of the effects of invasion on biodiversity and ecosystem function (e.g. the nature of the invasion density-impact curve, Yokomizo *et al*., 2009). Possible measures of invasion level include absolute or relative alien species diversity, abundance or impact. To decide which of these measures is most appropriate, attributes of a suitable indicator and the fundamental reason for interest in alien species invasion must first be considered.

### Attributes of a suitable indicator

The purpose of an indicator informs the type of metric that is selected, and selection inevitably involves trade-offs between desirable characteristics, measurement costs, value of information (Cairns *et al*., 1993) and the level of certainty vs. generality desired. Focusing on indicators as a means of assessing or detecting trends in invasion level and for use as early warning signs of degradation, we list the main criteria that should guide selection of invasion level indicators below (see Cairns *et al*., 1993; Boulton, 1999 and references therein):

1. Ecologically meaningful – ideally relates to the impact of aliens in the ecosystem, and should thus make good candidates on which to build a link between invasion level and invasion impact. We expand on this below.
2. Widely applicable and comparable – can be applied to a range of ‘response variable’ types (i.e. different types of organisms, individual species, groups of species and all species as a single group) and is comparable across ecosystems, biogeographic regions and spatial scales.
3. Independent of scale – because the temporal and, particularly, spatial scale at which invasion events are studied can vary, metrics that are independent of scale have more utility than those that are scale-dependent.
4. Measurable – can be clearly defined, relatively easy to measure accurately and precisely using a standard procedure, and should not require highly trained or experienced personnel to carry out the assessment.
5. Reliable – its response to a given level and type of change is predictable and consistent, so it conveys information that can be trusted. There is often a trade-off between the certainty and reliability of an index; the more general an index and the larger the spatial and temporal scale to which it applies, the more uncertainty it will introduce.
6. Interpretable and unambiguous – the meaning of different levels of invasion are clear and based on a sound scientific understanding, and levels deemed problematic are distinguishable from those that are acceptable. The indicator reflects ‘impacts’ of alien species and is not be confounded with effects of native biota or biogeochemical processes.
7. Cost-efficient – is relatively inexpensive and quick to measure.
8. Nondestructive of the ecosystem or native biota (though simultaneous control and measurement of alien species could be beneficial and cost-effective).
9. Repeatable through time – measurements taken at different points in time enables temporal changes to be detected.
10. Data availability – indicators that are built on data that is widely collected and widely available (i.e. currently and will be in the future) enable comparative studies and examination of trends through space and time.
11. Integrative and nonredundant – encompasses, but does not overlap, information provided by other variables.
12. Anticipatory – can act as an early warning of future degradation before serious impacts have occurred.
13. Timely – provides information rapidly that can be used to assess condition of ecosystem in a timely manner.

### Selecting ecologically meaningful indicators

Gaining knowledge and understanding to help alleviate impacts of human-mediated biological invasion is arguably the primary motivation for invasion ecology research (though the search for insights into community ecology is also key, Shea & Chesson, 2002). The **impact of invasion** at a particular location can be conceptualized as the product of the abundance or population density of alien species and their per capita effects (Parker *et al*., 1999; especially effects on resource availability: Shea & Chesson, 2002). However, species’ effects are highly variable (Gómez-Aparicio & Canham, 2008; Vilà *et al*., 2011) and are usually unknown (Vilà *et al*., 2010). Effects of invasion are notoriously difficult to quantify and qualitative assessments based on expert opinion should be avoided. Rather than relying on notions of harm (which are typically based on value judgments: Davis, 2009) or attempting to integrate multiple measures of effects (e.g. competition with native species, change in ecosystem structure), it is preferable to use biogeographic or ecological criteria for definitions and indices in invasion biology (Rejmánek, 2000; Valéry *et al*., 2008; Wilson *et al*., 2009). Consequently, the more objective and easier-to-quantify metrics of occupancy and abundance have typically been used to gauge invasion level (Lonsdale, 1999; Stohlgren *et al*., 2003; Chytrý *et al*., 2008a; Catford & Downes, 2010; Catford *et al*., 2011a). Although data on occupancy and abundance are generally widely available, these measures are not without limitations: data about recently naturalized alien species may be lacking, and occupancy and abundance may be underestimated due to issues such as poor detection (Garrard *et al*., 2008; Regan *et al*., 2011).

## Potential indicators of invasion level

### Ways to quantify invasion level

#### Binary invasion indices

The presence or absence of invasive species is a conservative way to determine whether an ecosystem is considered ‘invaded’ or not. However, the presence of a single individual of an invasive species is very different from an ecosystem that is dominated by invasive species, i.e. even if a species is classified as invasive, all sites where it occurs should not necessarily be declared ‘invaded’. This measure also relies upon correct classification of species as invasive or noninvasive; it may be beneficial to have a measure of invasion that is independent of measures of invasiveness of individual species.

Threshold values of alien species richness and abundance could be used to create binary variables that indicate an invaded ecosystem (Table 1). However, findings about important levels of alien species abundance vary widely (Gómez-Aparicio & Canham, 2008; Griffen & Byers, 2009), and it is unrealistic to think that a universal threshold value – or even categories of invasion level – exist. A scheme that ranks ecosystems according to their level of invasion depends on the set of ecosystems used and can be insensitive to relativities within the set of ecosystems considered.

**Table 1:** Advantages, disadvantages and utility of 12 potential indicators of ecosystem invasion level. Recommended indicators are marked with an asterisk; interpretation relates to both ecology and management unless specified; suggestions about ways that the indicator could be used for ecology or management are provided as examples only

Indicator	Comment	Advantages	Disadvantages	Interpretation	For ecology, indicates:	For management, indicates:
*Binary or categorical invasion scores*						
Presence/absence of alien species	May only be useful if representative alien species are used	Simple; information easy to gather and usually widely available	Provides little information; does not discriminate between level of invasion; most, if not all, ecosystems will have at least one alien species present	Absent = desirable	NA: insufficient information provided	NA: insufficient information provided
Threshold/s of alien species richness	Absolute or relative richness; e.g. if >10% richness = alien, then site considered invaded	Simple; richness data relatively easy to gather and usually the most widely available type of alien species data	Suitable threshold will vary among ecosystems, species and stage of invasion; setting threshold objectively require a lot of (often unavailable) information, so threshold will usually be subjective and based on expert opinion	Below threshold = desirable; categorical levels: lower alien richness = desirable	Ecosystems and sites with higher alien richness than others	Ecosystems and sites where alien species richness is considered to be of concern; categorical levels help to prioritize type of intervention, e.g. low richness – species-specific eradication and control, like herbicide application and biological control; high richness – ecosystem-level management
Threshold/s of alien species abundance	Absolute or relative abundance; categorical scores often used as a management assessment tool (e.g. habitat hectares, Parkes *et al*., 2003)	Simple; abundance data are relatively easy to gather, may not rely so heavily on detailed taxonomic knowledge (as is the case with richness) and are often available	Same as threshold of alien richness; data about alien species biomass, density and number of individuals are not widely available and can be hard to gather	Below threshold = desirable; categorical levels: lower alien abundance = desirable	Ecosystems and sites with higher alien abundance than others	Ecosystems and sites where alien cover is of concern; categorical levels of alien abundance help prioritize management actions, e.g. low cover – monitor, eradicate, protect; moderate cover – control and contain; high cover – contain
*Continuous invasion scores*						
Alien species richness	Number of alien species present in an ecosystem or site	Only requires information about the occupancy and distribution of alien species; positively correlated with number of invasive species	Interpretation not straightforward and can differ for ecology and management; does not provide information about relative contribution of alien species to the community; affected by spatial resolution of assessment	Low = desirable for management but not necessarily for ecology	High alien richness can indicate high richness of (undesirable) invasive species, but it may also indicate high habitat heterogeneity and functional diversity; can indicate degree of species saturation in community	Number of species that require management (targeted or otherwise)
*Relative alien species richness	Alien richness as a proportion of community richness	Accounts for species richness of invaded community; independent of spatial scale	Does not necessarily relate to alien cover or impact	Low = desirable	Potential for biotic homogenization, i.e. where indigenous biota is ‘diluted’ to such a degree that the community becomes more similar to other communities; can indicate resource availability and presence of ‘empty niches’ that are vulnerable to invasion; can indicate degree of species saturation in community	Potential for ecosystem recovery (low = better chance of recovery)
*Relative alien species richness for different functional groups		Indicates contribution of alien species to different structural components of a community; can potentially indicate if alien species represent a new functional group that may disrupt ecological networks and change biotic interactions	Functional group classification must be ‘fit for purpose’	Low = desirable; exceptions may arise in novel ecosystems when ecosystem function and services rely on alien species, and where a selection of alien species help to resist invasion by other species	The presence of empty niches in the invaded community; importance of species functional characteristics in determining invasion outcomes; potential changes in ecosystem structure and function	Potential for management actions to target particular functional groups; potential changes to ecosystem function and services
*Relative richness of transformer species	Relative contribution of transformer species to community richness	Indicates potential likelihood of ecosystem being altered by group of alien species; high richness indicates potential for high future abundance	Does not include species not yet recognized as transformers; provides no information about type of ecosystem transformation	Low = desirable	Systems vulnerable to invasion by transformer species; ecosystems at risk of abiotic and biotic change	Ecosystems at risk of abiotic and biotic change; management strategies must account for presence and effects of transformers
Alien species total abundance	Number of alien species present in an ecosystem or site	Helpful for determining scale of required management intervention	Provides no information about relative contribution of alien species to the community; affected by spatial resolution of assessment	Low = desirable	Level of resources available for alien species	Absolute magnitude of invasion; resources required and potential for control and eradication
*Relative alien species abundance	Alien abundance as a proportion of total community abundance	Accounts for native species abundance; value not affected by spatial resolution of study	Does not necessarily reflect effect and impact of alien species	Low = desirable	Level of resources available for alien species; can indicate presence of ‘empty niches’ that are vulnerable to invasion; high relative abundance and low richness/evenness may indicate potential for native species extirpation and reduction in diversity; can help gauge ecosystem invasibility	Potential for ecosystem recovery; affects type of control methods that can and need to be used (e.g. targeted mechanical removal and herbicide application vs. ecosystem-level approaches)
*Relative alien species abundance for different functional groups	Relative contribution of alien species to functional groups of interest	Indicates contribution of alien species to different structural components of a community; can indicate if alien species dominate a functional group, which may relate to their ability to disrupt ecological networks and alter biotic interactions		Low = desirable; exceptions may arise in novel ecosystems when ecosystem function and services rely on alien species, and where a selection of alien species help to increase ecosystem resistance to invasion by other species	The presence of empty niches in the recipient community; importance of alien species functional characteristics in determining invasion outcomes; potential changes in ecosystem structure and function: can indicate advent of novel ecosystems	Potential for management actions to target particular functional groups; potential changes to ecosystem function and services: can indicate advent of novel ecosystems
*Relative abundance of transformer species	Relative contribution that known transformer species make to community abundance	Indicates potential likelihood of ecosystem being altered by group of alien species	Does not include species not yet recognized as transformers; does not provide information about type or extent of ecosystem transformation	Low = desirable	Systems vulnerable to invasion by transformer species; ecosystems at risk of abiotic and biotic change	Ecosystems at risk of abiotic and biotic change; management strategies must account for effects of transformers
Alien species diversity	Could also use genetic or functional diversity	Integrated measure of richness and abundance	Many indices and no consensus on which to use, which can inhibit inter-study comparisons and meta-analyses; interpretation can be difficult because of complicated calculations and because low diversity can result from high cover by a few species (dominance) or low cover by many species	High = desirable ecologically, but low diversity will be easier for management as it can be targeted to the (fewer) alien species present	High diversity can indicate if alien species are present in high numbers but contribute evenly to cover (i.e. no dominant species)	Low diversity potentially easier for management because control interventions can be targeted
Alien species diversity relative to native species diversity	Ratio between alien and native diversity (i.e. alien H to native H); could also use genetic or functional diversity	Independent of scale of assessment	Many ways to relate alien diversity with native or community diversity, potentially resulting in inappropriate comparisons among studies	Difficult; high relative diversity of alien species compared to natives is undesirable, but so is low diversity that results from dominance by few alien species	Alien diversity > native diversity potentially indicates a native community vulnerable to invasion either because there are too few native species or because they are competitively inferior (e.g. isolated islands)	Low relative diversity of alien species is desirable because management can be targeted
*Alien species evenness	Accounts for dominance of individual alienspecies; dominance = inverse	Interpretation easier than diversity; can indicate presence of dominant alien species	Same as alien diversity; cannot indicate invasion level alone – should be interpreted with richness or abundance measures	Ecological interpretation unclear: low abundance and high evenness may be desirable, but high abundance and high evenness indicate more established species from which future individuals can recruit; low evenness is easier for management as it targets the (fewer) alien species present	Presence of dominant alien species that may be functionally novel or experiencing favourable biotic interactions, e.g. enemy release; presence of empty niches susceptible to invasion; high relative abundance and low evenness may indicate potential for native species extirpation and reduction in diversity	Low evenness potentially easier for management because control interventions can be targeted, but may also indicate presence of highly invasive species (e.g. ecosystem engineer) that will be difficult to control

#### Continuous invasion indices

Continuous variables could be used in absolute or relative terms, and dealt with singly or integrated using a multivariate approach (Parker *et al*., 1999) (Table 1).

*Absolute alien species richness* represents the number of alien species present in an ecosystem. Correlations have been found between richness of all alien plant species and the presence of invasive or noxious alien plants (Rejmánek & Randall, 2004; Chytrý *et al*., 2012), so high alien richness is more likely to be associated with larger impacts of invasion. However, Rejmánek & Randall (2004) found that this correlation only occurred when alien species richness exceeded 200 species and impacts may differ depending on characteristics of the recipient community. For instance, the addition of 10 alien species to species-poor or species-rich communities will likely have different consequences. Rather than high alien species richness being negative, it may indicate high habitat heterogeneity, which will foster species coexistence and thus high native species richness as well (Melbourne *et al*., 2007). Indeed, the switch from a negative correlation between native and alien species richness at local scales to a positive correlation at regional scales has been attributed to increases in habitat heterogeneity (Stohlgren *et al*., 1999; Fridley *et al*., 2007).

*Relative alien species richness* (i.e. proportion of all species that are alien) accounts for variation in native richness. It is independent of sampling plot size, so comparisons among studies are more straightforward than comparisons of absolute richness (Chytrý *et al*., 2008a). In most cases, low scores would be considered preferable to high scores, but a low proportion of alien species can potentially have greater effects than a high proportion. Examples include dominant species that form mono-specific stands (Ridenour & Callaway, 2001), or invasion of highly diverse communities by a small number of dominant, invasive species (e.g. invasion of South African fynbos communities by alien woody species; Richardson & Cowling, 1992).

*Absolute alien species abundance* (cover, biomass, number of individuals, density) indicates alien species’ population size or productivity, which is relevant for alien species management. Like absolute richness, absolute abundance values suffer from scale dependence and limited comparability among habitats. Hence, it is likely that *relative alien species abundance* will have a stronger link to invasion impacts. Of abundance measures, cover is perhaps the easiest to assess for plants and density for animals.

Measures of *diversity* and *evenness* (relative or normalized diversity: Kvålseth, 1991) provide an integrated measure of species richness and abundance. They are based on proportional abundance of each species relative to the total number of species (and their abundances) present in a community (Peet, 1974). Diversity and evenness (or reciprocally, dominance) provide useful information about the relative dominance of species, and can thus indicate whether an ecosystem is invaded by e.g. a single dominant species or several species whose cumulative cover equates to that of a single strong invader. As a result, diversity indices can be more informative than richness and abundance in isolation because they account for species’ evenness. However, this information is less readily available than cover and richness data (especially if sourcing data from the literature), and it can be difficult to interpret findings as diversity and evenness are often calculated in complex ways. Further, because there are various methods for calculating diversity (e.g. Simpson, Shannon, Berger-Parker, Rényi entropy, odds measure of diversity or – its inverse – homogeneity) and evenness (e.g. Simpson's evenness, Simpson's dominance, Pielou's evenness), and no consensus on which to use (Peet, 1974; Kvålseth, 1991; Smith & Wilson, 1996), care must be taken when comparing results among studies.

### Response variable of interest

In the preceding discussion, we have largely referred to all alien species, which includes those that are casual, naturalized but noninvasive, and naturalized invasive (Richardson *et al*., 2000; Milbau & Stout, 2008; Blackburn *et al*., 2011; Richardson *et al*., 2011). **Naturalized species** are alien species that survive and reproduce in their new range and sustain self-replacing populations without direct intervention from humans (Richardson *et al*., 2000). However, invasion indices (e.g. those in Table 1) need not be calculated from the entire suite of alien species present in a location, but could be based on a subset of alien species that are deemed representative or that can serve as ‘warning lights’ of invasion.

#### All alien species or only invasive alien species

Based on the ‘tens rule’ (Williamson, 1993), only 0.1 of naturalized species become invasive and cause impact. The tens rule is, to some extent, an artefact of the introduction history and residence time (Pyšek & Richardson, 2006), but even if all species in a lag phase were included, the majority of alien species will still be noninvasive (Ricciardi & Kipp, 2008). This is illustrated in Australia: 2739 of 26 242 alien plant species are currently classified as invasive and a further 5907 of these are predicted to become weeds in the future (Randall, 2007). The majority of alien species will thus be of little ecological or economic importance (Valéry *et al*., 2008), providing an argument for narrowing the scope to include only those that are actually invasive.

Studying invasive alien species increases understanding of factors that are influential across all stages of invasion and whether impacts of particular alien species can be ameliorated or managed. However, invasion is temporally dynamic (Milbau *et al*., 2009) and species must pass through several stages before becoming invasive (Richardson *et al*., 2000; Catford *et al*., 2009; Blackburn *et al*., 2011). Although some weed risk assessment procedures are now being widely adopted and perform well (Pheloung *et al*., 1999; Gordon *et al*., 2008), the criteria used to classify invasive and noninvasive species often vary among scientists, management organizations and jurisdictions, and identifying invasive species *a priori* can be difficult. Concentrating solely on species that are currently invasive will provide little insight into factors that affect species that are in a lag phase (Kowarik, 1995; Richardson & Pyšek, 2006). This may limit the ability to highlight ecosystems that actually do (or will) have high levels of invasion (or experience large effects of invasion).

Highly invasive species typically occur in systems that have high numbers and a high proportion of alien species (Rejmánek & Randall, 2004; Chytrý *et al*., 2012), so indices based on the full cohort of alien species will likely encompass the trends that would be found in metrics restricted to invasive alien species. As alluded to earlier, plant species from the ‘100 worst European list’ are more common in regions with higher alien species richness than in areas with lower richness (Chytrý *et al*., 2012), and the total number of naturalized and casual species has been found to reliably predict the number of noxious invasive species in the 50 US states (Rejmánek & Randall, 2004) [**casual alien species** do not form self-sustaining populations but rely on repeated introductions for long-term persistence (Richardson *et al*., 2000)]. This relationship could be used to predict the course of invasion: even if alien abundance is currently low, ecosystems rich in alien species will likely include dominant invasive species, so alien abundance may increase dramatically with time.

Some casual alien species can also affect ecosystems (Case, 1995), and using all alien species as a response variable removes the problems (e.g. bias, lack of data) associated with selecting which species to define as invasive. Although richness measures give invasive and noninvasive species equal weight, invasive species will be accorded higher leverage in measures of abundance or dominance because invasive species are typically more abundant than noninvasive ones.

#### ‘Native invaders’ or just alien invaders

There has been debate about whether native species (i.e. those that are locally indigenous) that have spread beyond their natural range and population density should be considered ‘invasive’ (Valéry *et al*., 2008; Catford *et al*., 2009; Richardson *et al*., 2011). While colonization and establishment processes are similar (Davis *et al*., 2000; Meiners *et al*., 2004) and arguments for not considering native species invasive some may consider weak (Valéry *et al*., 2008), we strongly advocate the use of **invasion** (and **invaders**) exclusively in the context of alien (nonindigenous) species whose presence in a region is attributable to human actions that enabled them to overcome fundamental biogeographical barriers. As well as evolutionary differences and associations with humans that set alien invaders apart from native ‘encroaching’ species (Mitchell *et al*., 2006; Kelly *et al*., 2008), this is a pragmatic approach. It is easier and requires less temporal information about species distributions (which is often limiting: Parker *et al*., 1999) to identify when alien species have expanded beyond their natural range than it is for native species that ‘naturally’ occur in the region.

#### Functional groups

The proportional abundance, richness and diversity of alien species for various growth forms, trophic levels or functional groups will indicate the relative contribution that alien species make to different structural components of a community. By changing ecosystem structure, addition of a new functional group is likely to have much larger consequences for ecosystem function than the addition of species that only differ from native species in the values of their traits (e.g. growth rates, body mass/size). For example, the ecological significance of invasive alien tree species in vegetation types with low native tree cover in parts of South Africa (Moll *et al*., 1980; Richardson & Cowling, 1992; Le Maitre *et al*., 2002) would be highlighted if proportional cover of the tree growth form was applied as an indicator. Invasion of novel predatory mammals in New Zealand is a similar case in point (Massaro *et al*., 2008), as are cats on islands (Medina *et al*., 2011). Where relative abundance is concerned, a group-based approach will also partially account for differences in the physical size of taxa that would otherwise make species’ abundances incomparable (Parker *et al*., 1999).

A shift in the relative abundance or diversity of different functional groups before and after invasion could also be used to gauge invasion level and the advent of novel (no-analogue) ecosystems, which may arise as a response to environmental change or as a direct consequence of alien invasion (Hobbs *et al*., 2006; Walther *et al*., 2009). Information about the condition of an ecosystem before invasion is often not available. However, this can partly be overcome by using a space-for-time substitution and comparing invaded sites with nearby sites that are not invaded but share the same environmental conditions (Holmes *et al*., 2000; Hejda *et al*., 2009).

#### Transformer species and ecosystem engineers

**Transformer alien species** that alter the character, condition, form or nature of an ecosystem over a broad area (Richardson *et al*., 2000) warrant special attention. While they may only comprise about 10% of invasive species (Richardson *et al*., 2000), such transformer species (or ecosystem engineers) can reduce local diversity, alter ecosystem structure and function, and modify disturbance regimes (Vitousek *et al*., 1987; Byers *et al*., 2010). The impacts of transformer species are varied (e.g. transformer plant species can stabilize or destabilize soil, promote or suppress fire and excessively use or provide limiting resources: Richardson *et al*., 2000), so there does not seem to be a standard way in which their effects can be reported. Instead, it would be informative to report the contribution that known transformer species (as a group) make to community abundance and richness. This of course will not necessarily correspond with the type or severity of impact caused by these species, nor will it include alien species that are yet to be recognized as transformers, but it will provide a general indicator of their presence and relative abundance. Examining temporal patterns of community richness and functional diversity relative to alien transformer species’ abundance may indicate the impact that transformer species have on local diversity.

#### Single species to represent invasion

If using a single species as an indicator of invasion, transformer species or invasive species that are considered noxious could be used to indicate the ‘worst-case scenario’ or to act as ‘warning lights’ of invasion. However, lag phases can limit recognition and detection of these species, and listed noxious alien species often reflect socioeconomic and political values rather than ecological ones (Parker *et al*., 1999) (though a correlation between economic and environmental impacts of alien plants has been found; Vilà *et al*., 2010).

A more objective and informative approach would be to select species that represent the broader alien species pool. This could involve choosing species based on their position in multivariate functional trait space relative to other alien species (Funk *et al*., 2008). Using a collection of species that encompass the variability of the whole alien species pool (i.e. species that occur at different positions, including the edges, of alien species trait space) would ensure that, of the traits considered, the functional range of alien species is included. In this approach, functional traits would not be used to identify traits associated with invasiveness, but rather as a means for generalizing across alien species.

### Spatial and temporal scale

The spatial extent at which invasion level is assessed should correspond with the boundaries of the ecosystem of interest, and the spatial resolution must be relevant to the organisms in question (Wiens, 1989).

As highlighted by the prevalence of lag phases (Kowarik, 1995; Richardson & Pyšek, 2006; Essl *et al*., 2011), the time at which invasion level is assessed is also crucial. As noted above, each species passes through a series of stages before becoming invasive (Richardson *et al*., 2000; Catford *et al*., 2009; Blackburn *et al*., 2011) and their collective stage will determine the stage of invasion that an ecosystem is experiencing. While populations and communities are affected by species at all stages of invasion, effects at the ecosystem-level are most apparent in later stages of invasion (Pyšek & Richardson, 2010; Vilà *et al*., 2011).

The rate and direction of change in invasion level can also vary. Even if two ecosystems have the same level of invasion at one time, they can have quite different futures depending on the identity of the alien species present, future propagule pressure, and the biotic and abiotic characteristics of the ecosystems. The value of standardized metrics is that trajectories of change can be plotted (potentially using space-for-time substitutions) to reveal the dynamics of the invasion process (e.g. linear increase through time, thresholds of invasion). Among other things, this will help to illuminate the course of invasion with respect to disturbance and succession (Rejmánek, 1989), and the opportunities that arise from fluctuations in resource availability (Davis *et al*., 2000) and invasion by other alien species (e.g. trophic cascades, invasional meltdown; Simberloff & Holle, 1999; White *et al*., 2006).

## Recommended invasion indices and their interpretation

This paper aims to establish standard approaches to quantify invasion level that enable meaningful comparisons among studies, ecosystems and regions. Of the 13 criteria we identified to assess the suitability of ecological indicators (described above, Cairns *et al*., 1993; Boulton, 1999), the first three are most important for meeting our goal: indicators must be (1) ecologically meaningful, (2) widely applicable and comparable and (3) independent of scale. Taking a decision tree approach, the three binary variables (presence/absence, thresholds of alien species richness and abundance) mean little for invasion level (and impact), so can be discarded. Diversity and evenness indices do not meet the second criterion because calculation methods are inconsistent across studies. Functional group classifications vary across taxa and systems (e.g. plants vs. animals, alpine tundra vs. tropical forest) and depend on the functional response of interest (e.g. grazing response vs. growth form). Identifying transformer species that modify ecosystem characteristics is difficult, and definitions of ‘transformation’ vary, so indices based these species are not widely comparable. Of the four remaining indices, alien species richness and abundance are not independent of spatial scale, which leaves relative alien species richness and relative alien species abundance.

We recommend the use of relative alien species richness and relative alien species abundance. These metrics meet the three criteria above, as well as the others considered: they are independent of scale, can be applied to a range of ‘response variable’ types and are comparable among regions and ecosystems, they offer certainty and reliability, are relatively easy and cheap to measure, are repeatable through time, their meaning is clear and interpretation relatively straightforward, data on them are widely available and they seem to encompass information provided by other variables. While work needs to be done, there is good reason to think that these indices relate to ecological impacts of alien species, making them suitable candidates on which to build a link between invasion level and impact.

The relationship between relative richness and abundance can indicate sites and ecosystems where relative alien cover is above that expected based on relative alien richness. Even without calculating species evenness, such information can indicate the presence of dominant alien species in a community (i.e. relative abundance > relative richness). These metrics can also identify ecosystems and sites that are heavily invaded or especially susceptible to invasion, identify sites at different stages of invasion, and indicate the trajectory of invasion over time.

The correlation observed between alien species richness and alien species abundance (Ortega & Pearson, 2005; Chytrý *et al*., 2009; Catford *et al*., 2011b) suggests that, if required, one of these metrics could be used in isolation. Data on species’ occupancy is generally more widely available and easier to collect than information about abundance, but the latter arguably reflects ecosystem-level impacts more accurately. In a study in bunchgrass communities of western Montana, USA, Ortega & Pearson (2005) found that, as a group, ‘strong’, noxious invaders (those known to dominate natural communities and displace native species) dominated alien cover, but not alien species richness (i.e. most alien species were ‘weak’ invaders, not strong ones). Correspondingly, under high levels of invasion (indicated by alien richness, cover and presence of *Centaurea maculosa*) native richness was negatively related to alien species cover but was not significantly related to alien richness. Cover of native species also decreased as cover of the most dominant invader (*C. maculosa*) increased (Ortega & Pearson, 2005). Although relationships between alien species abundance and impact are often nonlinear (Yokomizo *et al*., 2009), abundance measures incorporate the relative dominance of different invaders. Given that strong invaders are typically more abundant than weak invaders (Ortega & Pearson, 2005 and references therein), dominant invaders have greater leverage in measures of alien abundance (Williamson, 1993; Ortega & Pearson, 2005).

While metrics that quantify impacts of invasion may be more useful than those based on species occupancy and abundance, the types and significance of impacts vary from species to species and ecosystem to ecosystem making it difficult (and potentially impossible) to identify a standard and general index of invasion impact. Although there are limitations with using abundance and richness, these can be measured easily across all ecosystems and, when used in combination, they provide useful information. Determining the contribution that alien species make to different functional groups in a community, and establishing the richness and abundance (absolute or relative) of alien transformer species should help to indicate the current or potential impact of alien species on the ecosystems they invade.

Early action is crucial for managing invasive species efficiently, so indicators that are anticipatory and timely will be particularly important. However, the presence of some alien species in an ecosystem is more or less inevitable. Pragmatically, therefore, it seems appropriate to recommend that some threshold of invasion should be passed before a site can be flagged as a priority for management. Management-based objectives do not necessarily reflect ecological impacts of invasions; they encompass many other considerations (Rodríguez *et al*., 2007; Hobbs *et al*., 2009).

Single-species invasions may be easier to manage than multiple-species invasions because control and restoration can be targeted more effectively. However, if an ecosystem is heavily invaded or has been transformed by a highly invasive species, it is probably more effective to allocate limited management resources to less invaded ecosystems rather than highly invaded ones (Higgins *et al*., 2000). While high abundance (especially high proportional abundance) of alien species typically implies major influence and the potential to affect change, some alien species may help to achieve other environmental management objectives like ecosystem restoration (Ewel & Putz, 2004; Hobbs *et al*., 2009; Schlaepfer *et al*., 2011). Examination of the pattern and history of invasion levels (including the behaviour of particular alien species) in other sites and ecosystems can be instructive in setting management objectives. However, expert judgment is ultimately needed to determine the level and type of invasion at which management interventions should be implemented. This is particularly challenging in novel, or no-analogue, ecosystems that arise because of human-induced changes to the environment and biota; many invaded ecosystems are just such systems (Hobbs *et al*., 2006; Walther *et al*., 2009).

### Use of invasion level indices: application to two case studies

**Fig. 1:**
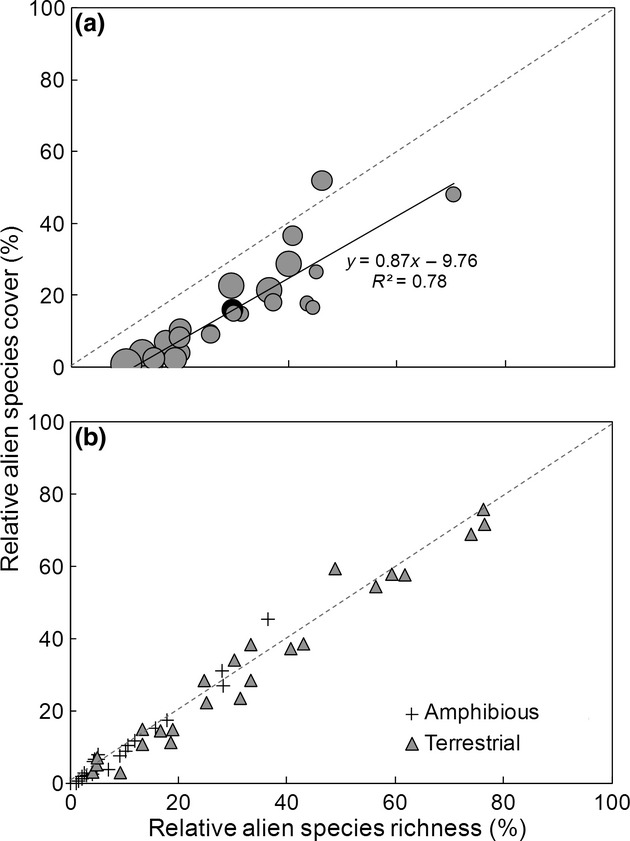
Level of invasion in 24 riparian wetlands illustrated by (a) relative richness, cover and dominance of all alien species, and (b) relative richness and cover of two functional groups – amphibious and terrestrial alien species. (a) Size of the circles indicates Simpson's dominance index calculated using alien species only (range: 0.10–0.54; larger circles indicate wetlands where alien species cover is dominated by fewer species); black circle is the mean level of invasion of all wetlands; black line is the line of best fit. (b) Richness and cover were calculated as a percentage of the total for each functional group; line of best fit for alien amphibious species, *y* = 1.13*x* − 1.06, *R*² = 0.97; line of best fit for alien terrestrial species, *y* = 0.98*x* − 1.05, *R*² = 0.97. Dashed grey line in both panels is the unity line. Wetlands were located along a 395 km reach of the River Murray, south-eastern Australia; for description of data, see Catford & Downes (2010).

**Fig. 2:**
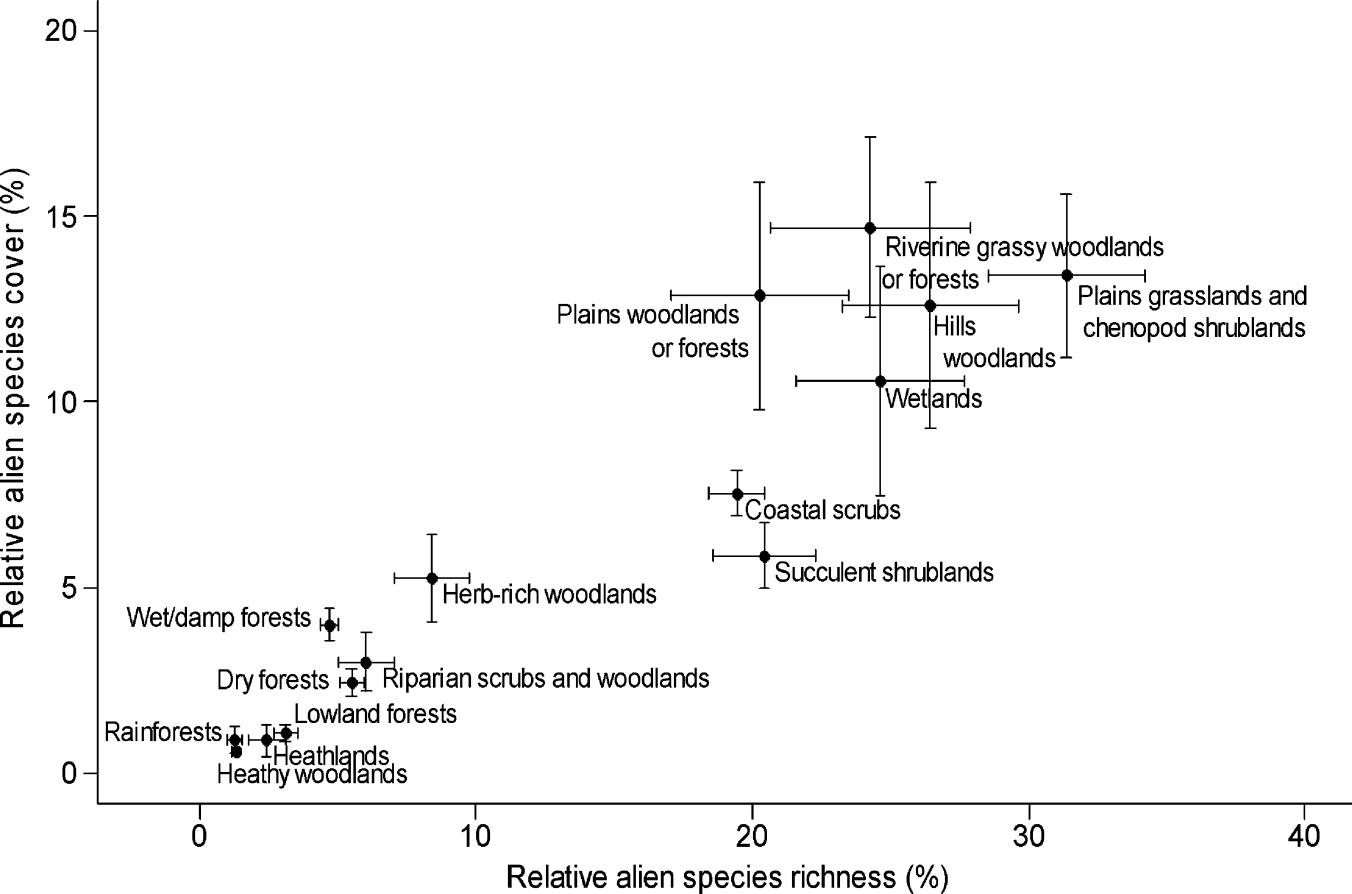
Level of invasion in 15 habitat types in the 13 340 km² Corangamite catchment in Victoria, Australia. Mean relative species cover is plotted against mean alien species richness (standard errors are shown). Data were gathered from 2717 vegetation plots (30 × 30 m) between 1972 and 2006 by the Victorian Department of Sustainability and Environment; for description, see Catford *et al*. (2011b).

We provide two case studies to illustrate the utility of our recommended indices for gauging ecosystem invasion levels. The first case study centres on 24 floodplain wetlands of the River Murray, south-eastern Australia (Fig. 1, Catford & Downes, 2010), and the second uses data from 2717 sites located in 15 different types of ecosystems, also in south-eastern Australia (Fig. 2, Catford *et al*., 2011b). Both case studies use floristic data to calculate relative alien species richness and relative alien species abundance, but the first also uses information about species evenness (Fig. 1a) and separates species into two functional groups (Fig. 1b). We do not distinguish between transformer and nontransformer species in these examples, but note that it can be done.

In the collection of wetlands used for the first case study, the line of best fit in Fig. 1a indicates that the relative contribution that alien species make to total vegetation cover is less than their contribution to total species richness, i.e. in terms of cover, alien species ‘punch below their weight’ in all but one wetland. Sites (or ecosystems) that are above the unity line should be of concern as the alien species present are contributing more cover than their native counterparts. Such an occurrence may indicate the presence of a strong, dominant invader in the community, which can be ascertained by examining specific information about species evenness. In this example, there is no strong pattern between invasion level and species’ dominance (Fig. 1a), and the evenness metric provided little additional information.

By separating species into groups based on their habitat preferences and life history strategies (i.e. amphibious or terrestrial wetland species: Brock & Casanova, 1997), more information is revealed (Fig. 1b). Richness and abundance of alien terrestrial species is generally greater than that of alien amphibious species (e.g. mean richness: 34.1% vs. 9.0%). However, the lines of best fit show that the slope for alien amphibious species is higher (1.13 vs. 0.98, both have a similar intercept, Fig. 1b). This suggests that, relative to their native counterparts, amphibious alien species were more dominant than terrestrial alien species.

The first case study illustrates that invasion levels can vary substantially within a single type of ecosystem in a defined geographic area. When comparing across ecosystems to gauge ecosystem invasibility, such variability should be considered (Fig. 2). The second case study shows how relative alien species richness and cover vary within and among 15 types of ecosystems. Overall, the invasion level of these ecosystems is below the unity line, but the large standard errors in several ecosystems (e.g. Plains woodlands, Hills woodlands) highlights that there are some sites that are above the unity line (Fig. 2). Variation can also indicate likely trajectories of invasion. For example, if a wetland in the study region currently has 25% alien species richness but only 7% alien species cover, it would be expected that the cover would increase to at least 11% in the future, bringing it on par with neighbouring wetlands (Fig. 2). To maximize management efficacy, it may be sensible to control alien abundance in this wetland before it increases and reaches a later stage of invasion.

As well as indicating ecosystem types that vary greatly in their invasion level, our second case study identifies ecosystems that experience comparatively higher levels of invasion overall. In this instance, a suitable management priority may be to limit future invasion in sites that are particularly susceptible to invasion (as indicated by their ecosystem type) but currently experience relatively low levels of invasion, e.g. sites in the Plains woodlands or forests ecosystem that currently have low relative alien species richness and cover.

## Calculating invasibility from invasion level: accounting for propagule pressure and invader traits

Characterizing ecosystem invasibility requires information about ecosystem invasion levels, propagule pressure and characteristics of introduced species. Fortunately, a strong research effort has revealed some key traits that make species invasive [e.g. high fitness, high growth rates and large size (van Kleunen *et al*., 2010), ability to outperform native species when resource availability is high (Daehler, 2003) and flexibility in niche requirements (Nentwig *et al*., 2010)]. This information could be used to quantify (and provide a comparable measure of) species invasiveness. For example, specific traits could be included as covariates in a regression model for predicting invasiveness. Attempts have already been made to develop general scoring systems that relate species’ traits with e.g. alien mammal impacts (Nentwig *et al*., 2010) and the distributional ranges and invasiveness of alien plant, insect and vertebrate species in Europe (Bacher *et al*., 2010).

The role of traits, however, is context-specific (Pyšek & Richardson, 2007). Among other things, the importance of species’ traits depends on the stage of invasion; species’ traits are most influential in later stages of invasion when species become invasive, whereas propagule pressure is crucial at the beginning of invasion (Pyšek *et al*., 2009). When inferring invasibility from invasion levels of ecosystems invaded by multiple alien species, traits may be less informative because many of the alien species present will be at early stages of invasion, i.e. a period when traits are weakly related to invasion level. Quantifying the invasiveness of individual species is also resource-intensive, and accurate information about species distributions may be lacking. Lack of knowledge about the nature of species’ interactions may limit the ability to adequately account for species invasiveness in calculations of invasibility. As well as interactions between alien and native species, alien-alien interactions will also affect ecosystem invasibility, i.e. alien species that invade first may potentially increase or reduce the invasibility of that ecosystem for subsequent invaders. Assessing the invasibility of a currently uninvaded ecosystem for a single alien species is relatively straightforward, but the problem becomes more complicated when multi-species invasions (i.e. the norm) are considered.

An alternative, albeit less rigorous, approach would be to exclude invading species characteristics from the calculations by treating all species as though they are equivalent, as has been done previously (Chytrý *et al*., 2008a; Catford *et al*., 2011b). However, the number of species that have had the opportunity to invade (i.e. colonization pressure: Lockwood *et al*., 2009) should still be considered. Unsuccessful species introductions have seldom been recorded (Diez *et al*., 2009), so accurate information about colonization pressure is usually lacking. However, including information about alien species richness will help provide this information to some extent, especially if species can be divided into invasive, naturalized and casual. For example, if the majority of alien species in one system are casual, whereas they are mostly naturalized and invasive in another, it could be inferred that the second system is more invasible. Correlations between the richness of naturalized/casual invaders and noxious ones (Rejmánek & Randall, 2004) suggest that including alien species richness in invasibility calculations would also partially account for the number of noxious species likely to be present in an ecosystem, which would affect invasion levels.

It is difficult to quantify propagule pressure directly, especially on a species-by-species level, so surrogates are usually used. These include national wealth, human population density, volume of ballast water discharge, metrics of human usage of particular species, proximity of roads and nearest city, and the density of urban, industrial or agricultural land (Pino *et al*., 2005; Herborg *et al*., 2007; Chytrý *et al*., 2008a; Pyšek *et al*., 2010; Castro-Díez *et al*., 2011). Depending on the taxonomic group of interest, it may be appropriate to use a single variable to indicate propagule pressure or to integrate several in a multivariate metric.

We do not attempt to integrate metrics of invasion level, invasiveness and propagule to calculate invasibility here: it is beyond the scope of this paper. However, we note that with knowledge about species invasiveness and propagule pressure, and with quantification of invasion levels across ecosystems, it should be possible to quantify ecosystem invasibility in a robust manner. From there, characteristics that affect ecosystem invasibility can be identified.

## Conclusions

There are multiple ways in which invasion level can be gauged. Selecting a suitable index for invasion level depends on the aims of research and management, and the two can differ both in their objectives and the way in which they interpret invasion scores. Because there are pros and cons of every metric, a suite of complementary indices might be required to meet the objectives of a single study or management initiative. We recommend the use of relative alien species richness and abundance (including alien species’ contribution to functional groups), which can be complemented with information about alien transformer species.
